## [Additional file 3. Review history. · Genome Biology]

Review history

First round of review

Reviewer 1

Comments to Author:

Review of vmeQTL in twins

In this study, the authors sought to identify vmeQTLs for blood DNA methylation variance using data from almost 1000 adult MZ twin pairs from two European twin studies. The analysis is very comprehensive, first with discovery in the TwinsUK study, with replication in the Netherlands twin register. Finally a number of confirmatory analyses are conducted. Together they provide evidence for vmeQTLs, many of which showed interesting disease associations. Overall the manuscript is very well written, it flows logically and easy to read despite describing multiple results. A very elegant and informative study.

I have a few concerns/requests to clarify some matters

1. Please provide more details on the twin samples which are a subset of the primary cohort. Are the analysed pairs all the MZ pairs with methylation data in each cohort, if not how were they selected for the present analysis.

How were the pairs originally selected for methylation analyses, i.e. were they chosen at random from the cohort's database or chosen on the basis of some traits or diseases.

Are the pairs included in the analysis representative of the entire UKTwins and NTR?

There is no information about the health status of the twins, yet both disease and treatment, including medications can affect methylation. Were any twins/pairs excluded on any basis exclusions. This is highly relevant given the interesting and novel associations with multiple immune system-related diseases. Could the associations have been driven by the presence of these conditions in a meaningful fraction of the twins?

What is known about the similarity of the twins for their external exposome, i.e. how many are living together or reside at the same or similar localities.

Are all pairs in the NTR of European Ancestry?

While they were all monozygous twins, the fetal environment of the two twins can differ substantially depending on the chorionicity. Is this information available and was it taken into account. The authors state that the prenatal environment is matched in MZ twins (page 7), but this is not always the case, which can manifest itself as large birth weight differences. Given MZ twins have unique methylation profile that is considered to have arisen early in the fetal period, what impact does that have on the findings of the present study?

Smoking classification was only current vs non-current smoking, where never and former smoker were combined. Smoking has a strong effect on DNA methylation, which is not reversed immediately upon quitting smoking. It would have been better to classify smoking status as never, former and current. Was smoking status validated against any biomarkers (CO, cotinine) in any subset of the twins in either cohort?

Was BMI based on measured weight and height, and was this measured at the same time as the blood draw for methylation analyses?

The following covariates were used: BMI, smoking, age and sex. Did you consider other potential covariates, why did you use only these?

Blood cell counts were inferred, was any validation against actual measurements done in any subset?

Minor:

Page 13, line 49 is the first occurrence of "MLR" , please spell it out here

What are the other phenotypes, diseases and exposures (Figure 4) page 19, line 46). Please expand or rephrase.

Page 19, line 58 you write " diseases, such as sarcoidosis, and neuropsychological traits including brain morphology and neuroticism"

I would not classify brain morphology nor neuroticism as neuropsychological traits

Page 34, next sentence is missing a word/words: The following probes were also removed: sex chromosomes, probes with a SNP within the CpG site (at the C or G position) irrespective MAF in the Genome of the Netherlands (GoNL) population, and ambiguous mapping probes reported by Chen et al with an overlap of at least 47 bases per probe[97].

Reviewer 2

The authors use variance effects on methylation as a path to discover gene environment interactions. The authors are clearly aware of the pitfalls of such an approach, where methylation QTLs can appear as variance effects and GxE at loci in partial LD, and use a novel test using monozygotic twins to get around this problem. The work is careful, and the conclusions justified, with a number of tests done on their analysis. The one issue that maybe is less addressed is the importance of scaling and normalisation when looking at variance and interactions, are the results robust to different choices?

Authors' response to reviewers

We thank the Reviewers for their comments on our manuscript. Please see below a detailed point-by-point response to the Reviewers' comments (in blue).

Reviewer 1:

Review of vmeQTL in twins

In this study, the authors sought to identify vmeQTLs for blood DNA methylation variance using data from almost 1000 adult MZ twin pairs from two European twin studies. The analysis is very comprehensive, first with discovery in the TwinsUK study, with replication in the Netherlands twin register. Finally a number of confirmatory analyses are conducted. Together they provide evidence for vmeQTLs, many of which showed interesting disease associations. Overall the manuscript is very well written, it flows logically and easy to read despite describing multiple results. A very elegant and informative study.

I have a few concerns/requests to clarify some matters

1. Please provide more details on the twin samples which are a subset of the primary cohort. Are the analysed pairs all the MZ pairs with methylation data in each cohort, if not how were they selected for the present analysis.

How were the pairs originally selected for methylation analyses, i.e. were they chosen at random from the cohort's database or chosen on the basis of some traits or diseases.

Are the pairs included in the analysis representative of the entire UKTwins and NTR?

We thank the Reviewer for this comment. We address the two related questions – “How were the pairs originally selected for methylation analyses” and “Are the pairs included in the analysis representative of the entire UKTwins and NTR” – together below:

DNA methylation data were generated for both monozygotic twins and dizygotic twins in both TwinsUK and NTR. The only criteria we used to select monozygotic twins for this study was to ensure that twins had available genotype and DNA methylation data, as well as age, sex, BMI, and smoking data. Given that the selection criteria focused on data availability rather than disease or health phenotypes, we believe that the samples included in this study are representative of both TwinsUK and NTR. We are only aware of 9 MZ twin pairs in the TwinsUK sample who reported to be discordant for disease.

We have added the following sentences in the Methods section (lines 694 to 700):

“TwinsUK is the biggest adult twin registry in the UK[55]. DNA methylation data were generated for both MZ and dizygotic (DZ) twins. The discovery stage involved the analysis of 355 pairs of European-ancestry female monozygotic twins who were not selected for particular diseases, with the exception of 9 pairs of twins discordant for rheumatoid arthritis [105]. Twins included in this study had available genotype data, as well as complete information on age, sex, BMI, and smoking from TwinsUK [92].”

We have also added the following sentences in the Methods section (lines 705 to 714):

“NTR is a population-based cohort of over 200,000 people from across the Netherlands[93]. It consists of twin-families, i.e. twins, their parents, spouses, and siblings aged between 0 and 99 years at recruitment, and started around 1987 with new-born twins, and adolescent and adult twins. DNA methylation profiles were derived for both MZ and DZ twins. We only included the MZ twins who have available genotype and DNA methylation data, age, sex, smoking, and cell component information. In families with multiple MZ twin pairs, we randomly selected one MZ twin pair to include. Individuals identified as non-European based on genotype principal component analysis (PCA) were excluded. As a result, we replicated the identified vmeQTL-vCpG associations in 633 pairs of MZ twins from the NTR, comprising 442 pairs of female twins and 191 pairs of male MZ twins.”

There is no information about the health status of the twins, yet both disease and treatment, including medications can affect methylation. Were any twins/pairs excluded on any basis exclusions. This is highly relevant given the interesting and novel associations with multiple immune system-related diseases. Could the associations have been driven by the presence of these conditions in a meaningful fraction of the twins?

To our knowledge at the time of collection only 9 MZ twin pairs out of 355 TwinsUK MZ twin pairs were discordant for an immune disease, specifically rheumatoid arthritis, based on self-reported questionnaires. Therefore, the majority of the sample were not selected for disease.

To explore this further, we also reviewed the available medication data collected by TwinsUK. We restricted the analysis to medication information collected no more than one month prior to blood collection, and only selected MZ twins with consistent self-reported medication information across all questionnaires collected, and for whom both co-twins had provided a response. We only had these medication data for 32 MZ twin pairs, and from these there were 9 twin pairs where one twin took medication that could potentially affect the immune system, for example, for allergy or asthma (see Table 1 below) – but their co-twin did not take this medication. These 9 twin pairs are distinct from the set of 9 RA-discordant MZ twin pairs.

Table 1. Medication data for 9 twin pairs from TwinsUK.

Family	Id	Medication Product	Medication Group
1	11	NA	Sleeping tablets; Inhaler for asthma;
1	12	NA	Anti-depressants; Drugs for malaria;
2	21	NA	Inhaler for asthma;
2	22	NA	Diuretic; Drugs for high cholesterol; Blood pressure drugs;
3	31	NA	Sleeping tablets; Steroids (oral inhaled or nasal);
3	32	NA	Drugs for high cholesterol; Drugs for angina (chest pain);
4	41	COLECALCIFEROL;LEVOTHYROXINE SODIUM (THYROXINE SODIUM);LEVOTHYROXINE SODIUM (THYROXINE SODIUM)	Calcium Carbonate;Levothyroxine Sodium;Levothyroxine Sodium
4	42	Levothyroxine;Calcium Carbonate;Simvastatin;Metformin Hydrochloride;Aspirin;Alendronic Acid	Thyroid Hormones;Compound Alginates&Prop Indigestion Prep;Lipid-Regulating Drugs;Biguanides;Antiplatelet Drugs;Bisphosphonates and Other Drugs
5	51	Co-Tenidone;Simvastatin;Co-Codamol	Beta-Adrenoceptor Blocking Drugs;Lipid-Regulating Drugs;Non-Opioid Analgesics And Compound Prep
5	52	SALMETEROL	Fluticasone Propionate (Inh)
6	61	Omeprazole;Cetirizine Hydrochloride;Nasonex	Proton Pump Inhibitors;Antihistamines;Drugs Used In Nasal Allergy
6	62	Lansoprazole	Proton Pump Inhibitors
7	71	Adizem-XL; Atorvastatin;Ezetimibe;Imdur;Temazepam	Calcium-Channel Blockers;Lipid-Regulating Drugs;Lipid-Regulating Drugs;Nitrates;Hypnotics
7	72	Amlodipine;Co-Proxamol;Furosemide;Isosorbide;Lorazepam;Simvastatin;Terbutaline Sulphate;Budesonide	Calcium-Channel Blockers;Non-Opioid Analgesics And Compound Prep;Loop Diuretics;Osmotic Diuretics;Hypnotics;Lipid-Regulating Drugs;Selective Beta(2)-Agonists;Corticosteroids
8	81	Tramadol;Metoclopramide	Opioid Analgesics;Drugs Used In Nausea And Vertigo
8	82	Prozac;Montelukast	Selective Serotonin Re-Uptake Inhibitors;Leukotriene Receptor Antagonists
9	91	Levothyroxine;Aspirin;Diclofenac Sodium	Thyroid Hormones;Antiplatelet Drugs;Non-Steroidal Anti-Inflammatory Drugs
9	92	Levothyroxine;Levothyroxine	Thyroid Hormones;Thyroid Hormones

We then repeated the vmeQTL analyses at the reported 359 vmeQTL-vCpG signals, by excluding these 18 MZ twin pairs – that is, the 9 RA-discordant pairs and the 9 pairs where one twin took medication that may affect the immune system, but the other did not. We observed that all 359 vmeQTL-vCpG associations remained significant in this subset of 337 MZ twin pairs (Figure 1).

Figure 1. Significance of 359 vmeQTL–vCpG associations in all 355 MZ twin pairs (X-axis) versus in 337 MZ twin pairs (Y-axis). Each dot represents a vmeQTL-vCpG association.

In summary, in the sample of 355 MZ twin pairs a subset of 18 MZ twins are either discordant for RA and or for use of medications that may affect the immune system, and these differences may lead to intra-twin-pair differences in blood cell composition. However, the 359 reported vmeQTL-vCpG associations remained significant when we exclude these 18 twin pairs from the analysis. We conclude that – based on the available self-reported data – this subset of twins does not have a major effect on the findings.

What is known about the similarity of the twins for their external exposome, i.e. how many are living together or reside at the same or similar localities.

In both TwinsUK and NTR, the majority of co-twins involved in the current study no longer lived in the same household at the time of blood sample collection.

To address this comment, we examined the most recent questionnaires in TwinsUK participants in this study: 208 pairs of MZ twins have available information, and of these 22 pairs lived together, and the distance between the 72 co-twin houses was within 10 miles based on postcodes.

Therefore, we conclude that a minority of the sample live together or very close together, and the majority are likely to have some differences in the external exposome. Both the UK and the Netherlands are relatively small and densely populated countries, and there will be some variation in the exposome especially with respect to urban and rural locations. A more detailed exploration of the living environment and exposome is beyond the scope of the current manuscript. For this first proof-of-concept study, we focus on variation in smoking, BMI, and cell components as possible drivers of the unique environment component.

We now add to the Discussion section (line 595 to 606)

“Furthermore, there are additional confounders in our study, for example, aspects of the twins’ external environment, as well as their chronicity, for which we do not have reliable data. Previous studies have suggested that chronicity may affect DNA methylation levels in blood [107,108], and it is also possible that the MZ twins have shared hematopoietic stem cells during development [109]. Moreover, it is very likely that environmental exposures are reflected to some extent in the human methylome, but the current study is limited by the scope of available data to robustly capture and study such external environmental exposures over the life course. Additionally, the BMI measurement date and blood sampling date were not concurrent in TwinsUK, but they were contemporaneous in NTR. Our previous simulation study found that power of vmeQTL detection may be lower without regressing out covariates first [41]. Therefore, all of these potential confounders may reduce vmeQTL detection power.”

Are all pairs in the NTR of European Ancestry?

Yes, we excluded ancestry outliers based on genotype principal components to ensure that all twins included from NTR are of Dutch ancestry.

We have added the following statement to the Methods section (line 711 to 712)

“Individuals identified as non-European based on genotype principal component analysis (PCA) were excluded”

While they were all monozygous twins, the fetal environment of the two twins can differ substantially depending on the chorionicity. Is this information available and was it taken into account. The authors state that the prenatal environment is matched in MZ twins (page 7), but this is not always the case, which can manifest itself as large birth weight differences. Given MZ twins have unique methylation profile that is considered to have arisen early in the fetal period, what impact does that have on the findings of the present study?

We agree that the differences can arise in the prenatal environment, leading to variation between monozygotic twin pairs' DNA methylation profiles. Information on chorionicity is unfortunately unavailable for both TwinsUK and NTR samples. However, 193 of 200 identified vmeQTL-CpG associations were validated in non-twin individuals from the 1946 UK birth cohort. This suggests that the majority of the MZ twins vmeQTLs do not reflect vmeQTL-chorionicity interaction effects.

We now discuss the lack of information on chorionicity as a limitation in the Discussion section (line 595 to 606):

“Furthermore, there are additional confounders in our study, for example, aspects of the twins' external environment, as well as their chronicity, for which we do not have reliable data. Previous studies have suggested that chronicity may affect DNA methylation levels in blood [107,108], and it is also possible that the MZ twins have shared hematopoietic stem cells during development [109]. ... Therefore, all of these potential confounders may reduce vmeQTL detection power.”

Smoking classification was only current vs non-current smoking, where never and former smoker were combined. Smoking has a strong effect on DNA methylation, which is not reversed immediately upon quitting smoking. It would have been better to classify smoking status as never, former and current. Was smoking status validated against any biomarkers (CO, cotinine) in any subset of the twins in either cohort?

This point has been investigated previously in NTR (<https://elifesciences.org/articles/83286>). In that study, the authors stated, “We previously described smoking misclassification in this cohort based on blood levels of cotinine (van Dongen et al., 2018), a biomarker for nicotine exposure, that has been measured in a subset of the cohort (Bot et al., 2013), which indicated a low misclassification rate. Plasma cotinine levels were available for 591 individuals classified as never smokers by self-report. Five of these individuals (0.8%) had cotinine levels ≥ 15 ng/ml, which is indicative of smoking, and thus indicates a misclassification of smoking status.” These results indicate that the majority of individuals in the cohort have self-reported smoking status consistent with their blood cotinine levels.

We agree that the DNA methylation patterns in ex-smokers may not be the same as those of never-smokers. Therefore, we have conducted a sensitivity analysis to evaluate if smoking classification affects vmeQTL results. Using the same processing steps, but categorizing participants into three classes of smoking (current, ever, and never), we reran the vmeQTL-CpG associations. All 359 vmeQTL-CpG associations remained significant in the 3-smoking level model and p-values did not change substantially (Figure 2).

Figure 2. Significance of 359 vmeQTL–vCpG associations when comparing smoking status categorized into three groups versus two groups. Each dot represents a vmeQTL–vCpG association. This analysis was performed using 355 MZ twin pairs from the TwinsUK discovery cohort. Smoking status was classified as never, ever, and current (Y-axis), or as non-current (combining never and ever smokers) and current (X-axis). Intra-twin discordance or concordance in smoking status was then used as a covariate.

Based on these results, we conclude that our findings in this study are robust to smoking classification from 3-level to 2-level smoking status.

Was BMI based on measured weight and height, and was this measured at the same time as the blood draw for methylation analyses?

In the NTR cohort sample, BMI was based on measured weight and height obtained at the same time as the blood draw.

In the TwinsUK cohort sample, BMI was calculated using weight and height measured at the same time. Among the 355 MZ twin pairs:

- 125 pairs (38%) had the same BMI measure date and blood sample date,
- 134 pairs (38%) had a BMI measure date and blood sample date that were not exactly the same, but fell within a year,
- 82 pairs (23%) had a BMI measure date and blood sample date more than one year apart, and
- 14 pairs (4%) did not have this information.

Given that DNA methylation is associated with BMI changes, twin pairs with a longer interval between blood sampling and BMI measurement may not accurately reflect BMI effects on DNA methylation.

We now state in the Discussion section (line 595 to 606)

“... Additionally, the BMI measurement date and blood sampling date were not concurrent in TwinsUK, but they were contemporaneous in NTR... Therefore, all of these potential confounders may reduce vmeQTL detection power.”

The following covariates were used: BMI, smoking, age and sex. Did you consider other potential covariates, why did you use only these?

In addition to BMI, smoking, age, and sex, we also considered DNA methylation-derived cell components and batch effects. These are well-studied factors that have been shown to influence whole-blood DNA methylation levels. Therefore, we decided for this first proof-of-concept paper to focus on these factors as potential drivers of the unique environment component.

Blood cell counts were inferred, was any validation against actual measurements done in any subset?

In NTR, measured cell counts were available for the same sample. Using the Coulter system (Coulter Corporation, Miami, USA), a haematological profile was obtained from EDTA blood directly after the samples arrived in the laboratory. Parameters included white blood cell count (WBC), including percentages and numbers of neutrophils, lymphocytes, monocytes, eosinophils, basophils.

We assessed the correlation between measured white blood cell proportions (rows) and estimated white blood cell proportions based on the Houseman method (columns) in the sample of monozygotic twins from the NTR, with blood DNA methylation data available. Correlations between parameters representing the same white blood cell fractions were high (e.g. Table 2 granulocytes/neutrophils, $r=0.90$, monocytes, $r=0.71$), although not perfect. Imperfect correlation may reflect limitations of the size and representativeness of the reference population that was used to build the cell type deconvolution algorithm.

Table 2. Correlation in estimated and measured blood cell proportions from NTR sample.

	Cellular proportions, estimated (Houseman)								
Cellular proportions, measured	CD8T	CD4T	NK	Bcell	Mono	Gran	PlasmaBlast	CD8pCD28nCD45Ran	CD8.naive
Neutrophils	-0.49	-0.63	-0.26	-0.51	0.22	0.90	0.67	0.19	-0.32
Lymphocytes	0.52	0.67	0.23	0.53	-0.44	-0.88	-0.71	-0.23	0.36
Monocytes	-0.04	-0.06	0.14	0.01	0.71	-0.21	0.03	0.02	-0.06
Eosinophils	0.01	0.05	0.07	0.04	0.07	-0.10	-0.02	0.10	-0.03
Basophils	0.02	0.04	-0.05	-0.01	0.02	-0.01	0.01	0.00	-0.03

The rationale for using the inferred cell components in this study is that measured cell components are not available in TwinsUK, but cell composition strongly impacts DNA methylation. Besides, estimated cellular proportions provide information on additional white blood cell subtypes that are not directly measured. Given the high correlation observed in NTR between estimated and measured cell components, we used Houseman’s algorithm estimated cell proportions in both TwinsUK and NTR to ensure consistency of covariates across cohorts.

We have added the above table to the Supplementary Tables as Table S9 and include the following text in the Methods section (line 774 to 790)

“Because measured cell counts are not available in TwinsUK, cell proportions were estimated by the Houseman et al[89] algorithm in both TwinsUK and NTR cohort samples. Cell estimates were obtained for plasmablasts, memory and effector T cells (CD8pCD28nCD45Ran), naïve CD8+ T cells, naïve CD4+ T cells, CD8+ T cells, CD4+ T cells, natural killer cells, B cells, monocytes, and granulocytes. To assess the performance of the blood cell proportion estimation, we compared the correlation between the estimated cell proportions and previously measured cell proportions from the same samples in NTR [106]. Briefly, in the NTR cohort, a haematological profile was obtained from EDTA blood using the Coulter system (Coulter Corporation, Miami, USA) after the samples arrived in the laboratory. Parameters provided included white blood cell count (WBC), with percentages and numbers of neutrophils, lymphocytes, monocytes, eosinophils, basophils. Correlations between parameters that reflect the same estimated and measured white blood cell fractions are high (Supplementary Table 9). For example, the Pearson’s correlation between estimated granulocytes and measured neutrophils is

0.90 and the estimated and measured monocytes correlation is 0.71 (Supplementary Table 9). To maintain consistency in covariates across cohorts, we used estimated cell proportions in both TwinsUK and NTR. ”

Additionally, we added to the Discussion (line 591 to 593):

“Although correlations between the estimated blood cell fractions using the Houseman algorithm

[89] and measured cell proportions are high, they are not perfect.”

Minor:

Page 13, line 49 is the first occurrence of "MLR" , please spell it out here We thank the Reviewer for pointing this out. We have now spelled out MLR as myeloid to lymphoid ratio (MLR) in line 261.

What are the other phenotypes, diseases and exposures (Figure 4) page 19, line 46). Please expand or rephrase.

To address this comment, we now state (line 408 to 411):

“We found significant enrichment for vCpGs to be EWAS signals for cardiometabolic, immune, and neuropsychiatric traits, as well as for diseases such as bladder cancer and cystic fibrosis, and for exposures such as malathion and household socioeconomic status in childhood (Figure 4).”

Page 19, line 58 you write " diseases, such as sarcoidosis, and neuropsychological traits including brain morphology and neuroticism"

I would not classify brain morphology nor neuroticism as neuropsychological traits

We have now rephrased this to state (line 416 to 417):

“... diseases, such as sarcoidosis, and multiple traits including neuroticism and aspects of brain morphology.”

Page 34, next sentence is missing a word/words: The following probes were also removed: sex chromosomes, probes with a SNP within the CpG site (at the C or G position) irrespective MAF in the Genome of the Netherlands (GoNL) population, and ambiguous mapping probes reported by Chen et al with an overlap of at least

47 bases per probe[97].

We thank the Reviewer for pointing this out. We have rephrased this sentence (line 760 to 765): *“The following probes were also removed: probes on sex chromosomes, probes with a SNP within the CpG site (at the C or G position) irrespective of MAF in the Genome of the Netherlands (GoNL) population, and ambiguously mapping probes reported by Chen et al (2013) with an overlap of at least 47 bases per probe [97]. The methylation data were normalized with functional normalization[98].”*

Reviewer 2:

The authors use variance effects on methylation as a path to discover gene environment interactions. The authors are clearly aware of the pitfalls of such an approach, where methylation QTLs can appear as variance effects and GxE at loci in partial LD, and use a novel test using monozygotic twins to get around this problem. The work is careful, and the conclusions justified, with a number of tests done on their analysis. The one issue that maybe is less addressed is the importance of scaling and normalisation when looking at variance and interactions, are the results robust to different choices?

We thank the Reviewer for this comment. To address this point, we tested whether the 359 vmeQTL-vCpG associations remain significant after scaling or normalizing the input data in the MZ twin model, using the 355 pairs of monozygotic twins in TwinsUK. We applied two approaches to normalise the data (min-max and rank-inverse normalization), and observed that the 359 vmeQTL-vCpG associations are robust to such data transformations.

We have added these new results to Supplementary Table 10 and Supplementary Note section 1.4, where we now state:

“1.4 Validation of vmeQTL–vCpG associations using different data normalisation and scaling methods

To assess whether scaling and normalisation affect the significance of the 359 vmeQTL-vCpG associations, we applied

min-max scaling and rank-based inverse normal transformation (RINT) to the intra-twin methylation difference residuals obtained after adjusting for covariates.

When using the min-max scaled data, all 359 vmeQTL-vCpG associations remained significant at the Bonferroni-corrected threshold ($P < 0.05/359$) (Supplementary Table 10). Similarly, using the RINT-transformed data, we observed that 358 out of 359 vmeQTL-vCpG associations surpassed the Bonferroni threshold (Supplementary Table 10). The remaining association (vmeQTL: 5:31796741_A_G, vCpG: cg17413084, $P = 2e-4$) exceeded nominal significance, but not the Bonferroni-corrected threshold. Therefore, although scaling and normalisation change the range and distribution of the data, they do not change the order of values. These results indicate that the 359 vmeQTL-vCpG associations are robust to such data transformations.”